# Impact of Living Alone on Depressive Symptoms in Older Korean Widows

**DOI:** 10.3390/ijerph14101191

**Published:** 2017-10-07

**Authors:** Gyeong-Suk Jeon, Kyungwon Choi, Sung-Il Cho

**Affiliations:** 1 Department of Nursing Science and Research Institute of Women, Mokpo National University, 1666 Yeongsan-ro, Cheonggye-myeon, Muan-gun, Jeonam 534-729, Korea; gsj64@naver.com or sookie@mokpo.ac.kr; 2 Department of Nursing, Korea National University of Transportation, 61 Daehak-ro, Jeungpyeong-gun Chungbuk 27909, Korea; 3 Department of Public Health Science, Graduate school of Public Health and Institute of Health and Environment, Seoul National University, 1 Gwanak-ro, Gwanak-gu, Seoul 08826, Korea; scho@snu.ac.kr

**Keywords:** living alone, depressive symptoms, widows, socioeconomic status, social ties

## Abstract

We examined the relationship between living alone and the prevalence of depressive symptoms in older Korean widows and assessed the individual contributions of health, social ties, and socioeconomic factors to the development of depressive symptoms. The study was a secondary analysis using data from widows, 65 years of age and older, who participated in the Living Profiles of Older People Survey (LPOPS). A logistic regression analysis was used to evaluate the contributions of health, social ties, and socioeconomic factors to the development of depressive symptoms. Working status and equivalent household income were significantly associated with depressive symptoms in both those living with others and those living alone. Adjustment for health status and social ties did not change the impact of living alone on the prevalence of depressive symptoms. However, adjustment for equivalent household income eliminated the negative association between living alone and depressive symptoms. Our findings indicate that economic resources are more important than health and social ties for alleviating the negative impact of living alone on the development of depressive symptoms in older widows.

## 1. Introduction 

Being widowed is a stressful life event that involves transitioning and adapting to new roles which are associated with negative stress [1]. Several studies have shown that older widows have more depressive symptoms than their counterparts [2,3,4]. Furthermore, the death of a spouse involves changes in living arrangements that may have an impact on the quality of life and mental health in older adults [5]. The number of older adults who live alone is increasing in developed and developing countries because of a steady rise in life expectancy and a gender gap in longevity [6]. Older women tend to live longer than their husbands; thus, older women are more likely to be widowed and live alone. According to the 2013 United Nations World Population Aging Report, almost half of the women who live independently live alone, whereas only a small proportion of older men live alone [6]. In Korea, the rapid increase in widows living alone has led to major demographic changes. In 2010, half of elderly widows reported living alone compared with less than 5% in 1960 [7].

Although living arrangements may be dictated by sociodemographic factors and underlying cultural norms and preferences [8], living alone is often seen as an undesirable state and considered to be a risk factor for mental health, similar to loneliness and depression [9]. However, the findings of previous investigations regarding the relationship between living alone and the prevalence of depressive symptoms in older adults are inconsistent. Population studies of older adults in the Netherlands, Hong Kong, Japan, and the United States have found that older adults living alone are more likely to have depressive symptoms and poor mental health than those living with their spouse, children, or others [2,10,11,12]. However, some authors have argued that living alone in later life was not in itself a strong risk factor for psychological distress [13]. For instance, some studies have shown that living alone decreased the frequency of angry feelings and emotional distress among older adults [14,15], and that older women living alone reported superior psychological functioning relative to those living with a spouse [16]. Additionally, previous studies have shown that elderly adults living in multigenerational households are more likely to experience depressive symptoms than those living alone because stress resulting from conflicts between family members may lead to depression [17,18]. 

The effect of living alone on the development of depressive symptoms may differ according to each individual’s condition; however, previous studies have shown that health status, social ties, and the socioeconomic status of widowed older adults have an impact on living alone [19,20,21,22]. Although these factors may underlie the variability in the association between living alone and depression in older widows, the impact of each variable on the development of depressive symptoms in elderly individuals living alone has not been sufficiently investigated. 

In Korean tradition, bereaved parents are expected to live with their adult children and consider it normal to get financial, practical, and emotional support from them [23]. Under the teaching of Confucianism, filial piety, called “hyo”, is an important social norm that influences the care-related decisions and practices of older adults and their children [24]. One of the filial responsibilities of “hyo” is to care for parents with compassion and altruistic concern [25]. Therefore, living alone may have a more detrimental effect on elderly Korean adults than those living in individualistically-oriented Western cultures because of cultural preferences for family closeness and co-residence. However, this family-oriented tendency is changing in Korea: the percentage of older adults living with their children decreased from 54.7% in 1994 to 28.4% in 2014 [26]. Moreover, according to the Korea Statistics survey [27], 75.1% of older Korean adults reported that they did not want to live with their adult children. These findings indicate that the filial norms in family ties associated with traditional Korean culture have weakened and that the cultural and social value of co-residence is changing. This may be shared in other Asian countries under similar influence of Confucianism and it may contribute to the prevalent depressive symptoms of the elderly population in East Asia [28]. 

Given that the changing social environment in Korea may influence the association between living alone and the prevalence of depression in widows, further study of this relationship in elderly Korean widows in contemporary society is warranted. Additionally, we were interested in the individual contributions of health, social ties, and socioeconomic factors to the development of depressive symptoms in older widows living alone. We believe our study will improve understanding of the complex nature of the relationship between living alone and the development of depressive symptoms in older widows. 

## 2. Materials and Methods

### 2.1. Design and Study Population

The data were drawn from the Living Profiles of Older People Survey (LPOPS) conducted by the Ministry of Health and Welfare in Korea. The LPOPS is comprised of 4356 men and 6095 women aged 65 years or older and living in communities in South Korea. Using a two-stage stratified cluster sampling method, older residents were selected from households stratified by 25 metropolitan and provincial (urban and rural) regions. Trained surveyors visited the participants at their places of residence and completed the full survey and written questionnaire and obtained informed consent. In view of the study aims, female participants who were married, divorced, separated, or never married (*n* = 3078) were excluded. Furthermore, widows with a physical disability (*n* = 131) were excluded because co-residence could not be avoided in these cases. Physical disability was measured using the Korean version of the Instrumental Activities of Daily Living scale (K-IADL) [29]. The K-IADL includes 10 questions about instrumental daily living, such as personal grooming, excursions for short distances, transportation use, making and receiving phone calls, managing money, performing household chores, preparing meals, shopping, taking medications, and doing laundry. Respondents who were dependent on others for one or more of the instrumental activities of daily living were considered to have a physical disability. After exclusions, a weighted population of 2799 widows aged 65 years or older with no disability was included in the final analysis.

### 2.2. Assessment and Measurements

*Depressive symptoms.* Depressive symptoms were measured using the Korean version of the Geriatric Depression Scale-Short Form (SGDS-K), which was developed by Sheik and Yesavage [30] and translated into Korean by Bae and Cho [31]. The SGDS is composed of 15 items comparable to the 30 items on the Korean version of GDS. The Korean versions of the GDS and SGDS are valid and widely used tools. A previous Korean community-based study identified the optimal cut-off point for screening major depressive disorder as a SGDS-K score of 8 or higher. The SGDS-K has satisfactory reliability (Cronbach’s alpha of 0.90) and validity [31]. 

*Number of chronic diseases.* The number of diseases was recorded when the participants reported one or more physician-diagnosed conditions, including cardiovascular disease (hypertension, stroke, hyperlipidemia or angina pectoris), endocrine disease (diabetes or thyroid disease), musculoskeletal disease (arthritis, osteoporosis, back pain, or sciatica), pulmonary disease (chronic obstructive pulmonary disease, asthma or tuberculosis), cancer, gastrointestinal disease (hepatitis or liver cirrhosis), genitourinary diseases (chronic renal failure, benign prostate hyperplasia, urinary incontinence or sexually transmitted infection), eye and ear disease (cataracts, glaucoma or chronic otitis media), and other diseases (anemia or chronic dermatologic disease). 

*Measure of social ties.* We used three variables to measure social ties: relationship with children, relationship with friends and neighbors, and social participation. Relationship with children was assessed by the question “How would you rate your relationship with your children?” The five response options were: 1 = very good, 2 = good, 3 = fair, 4 = bad, 5 = very bad. We dichotomized the responses to this question as “very good” or “good” versus the remaining categories. Older widows with no children were categorized as “no children”. Participants rated the quality of their relationships with friends and neighbors on a scale from 1 (very good) to 5 (very bad) in response to the question, “How would you rate your relationship with your friends and neighbors?” We dichotomized the responses to this question as “very good” or “good” versus the remaining categories. Social participation was judged by a “yes” response to participation in any of the following social activities: friendships, hobbies, leisure-time, or political society activities. A “yes” answer for any social activity was considered as involvement in a social participation group.

*Socioeconomic variables.* Three variables, educational attainment, working status, and annual household income, were used to determine socioeconomic status. Education levels were classified as middle school or higher, elementary school, and uneducated. Current working status was classified as “yes” or “no”. Annual income was used as a measure of household income. Total household income was divided by the square root of the number of household members and then categorized according to tertile distributions of all responses combined (lowest 33.3%; middle 33.3%; highest 33.3%). 

*Other covariates.* Age (65–74, 75–84, 85 or over), area of residence (urban or rural, and religion (yes or no) were included as covariates. 

### 2.3. Statistical Analysis 

The data are expressed as frequencies and means (± standard deviation [SD]) of the baseline characteristics, and between-group differences in health and social factors were compared using chi-squared tests (Table 1). We used a logistic regression analysis to assess the individual contributions of health, social ties, and socioeconomic factors to the development of depressive symptoms (Table 2). The reference model (Model 1) was used to estimate the odds ratios (ORs) of living arrangement for depressive symptoms, adjusted for age, area of residence, and religion. Health status was included and replaced by social ties and socioeconomic factors, one by one, in Models 2–8. For each model, we calculated the percentage change in ORs for depressive symptoms compared with the reference model (i.e., Model 1; [(OR_(Model 1)_ − OR_(Model 2–8)_/(OR_(Model 1)_ − 1) × 100]). The explained fractions (XF) for health status, social ties, and socioeconomic factors calculated from the ORs of depressive symptoms represented the contribution of differential exposure [32]. Multicollinearity among covariates was evaluated using correlation analyses based on the variance inflation factor and tolerance tests; no significant collinearity was detected between any covariates. All statistical tests were conducted using the Statistical Package for the Social Sciences software v. 22.0 for Windows (SPSS, Inc., Chicago, IL, USA). Our study was approved by the Ethics Review Board of the Mokpo National University, with which the researchers were affiliated (20170719-SB-014-01). 

## 3. Results

Participant characteristics, including chronic diseases, social ties, and socioeconomic status, as well as the prevalence of depressive symptoms according to living arrangement, are shown in Table 1. More than half of the participants (60.7%) lived alone. Those living alone were more likely to live in a rural area, have better relationships with their friends and neighbors, be working, and have a lower household income than elderly adults living with others. The percentage of participants with no children living alone was significantly higher than that of those living with others. 

The prevalence of depressive symptoms was higher in elderly adults living alone (24.3%) than in those living with others (20.6%). As expected, the chi-square tests revealed that the number of chronic diseases, relationships with children and friends/neighbors, social participation, working status, and equivalent household income were significantly associated with depressive symptoms in both those living with others and those living alone. However, age was significantly associated with depressive symptoms in elderly adults living alone, but not in those living with others. 

The results of the multivariate logistic regression analysis of the associations between depressive symptoms and living arrangement, health status, social ties, and socioeconomic factors are shown in Table 2. Model 1 included living arrangement and confounding factors (age, area of residence, and religion) as a baseline model. Elderly widows living alone had a higher likelihood of having depressive symptoms than those living with others (OR 1.24, 95% CI 1.03–1.49). In Models 2–8, health status (chronic disease), social ties, and socioeconomic factors were sequentially added to Model 1 to explore the impact of each factor on the odds of depressive symptoms for living alone. The magnitude of the association between living alone and depressive symptoms decreased slightly but remained significant in Model 2, which included having a chronic disease (OR = 1.22, 95% CI = 1.01–1.47). Moreover, having a chronic disease was independently associated with depressive symptoms. Model 2 revealed that having a chronic disease accounted for 1.27% of the difference between living alone and co-residency on the prevalence of depressive symptoms. Models 3–5 successively added each of the three social relationship factors to Model 1; relationship with children, relationship with friends/neighbors, and social participation accounted for 0.37, 5.91, and 4.92%, respectively, of the effect of living alone on depressive symptoms. All of the ORs for these variables in each model were statistically significant and greater than unity, indicating that poor social relationships increased the risk of depressive symptoms in older South Korean widows. In Models 6 and 7, the odds of an association between living arrangement and depressive symptoms (OR = 1.26, 95% CI = 1.04–1.51 for Model 6; OR = 1.28, 95% CI = 1.07–1.55 in Model 7) were slightly increased when educational attainment (1.52% in Model 6) and working status (3.73% in Model 7) were added to Model 1. Moreover, educational attainment and working status were independently associated with depressive symptoms. In Model 8, which included equivalent household income, the OR for living alone in relation to depressive symptoms decreased markedly, from 1.24 to 0.74, while equivalent household income accounted for 39.94% of the effect of living alone on depressive symptoms. Living alone was associated with a significantly lower risk of depressive symptoms compared with living with others (OR 0.74, 95% CI 0.60–0.93). Furthermore, equivalent household income was independently associated with depressive symptoms. When all of the variables were simultaneously included in Model 9, the OR of living alone for depressive symptoms decreased from 1.24 to 0.99, accounting for 19.79% of the association between living alone and depressive symptoms. 

## 4. Discussion

We investigated the impact of living alone on the prevalence of depressive symptoms in widows living in contemporary Korean society. Additionally, we assessed the explanatory value of various factors that may affect the association between living alone and the development of depressive symptoms. We found that adjustment for health status and social ties did not change the impact of living alone on the prevalence of depressive symptoms. However, adjustment for equivalent household income eliminated the negative association of living alone or had a positive effect on depressive symptoms. This financial factor accounted for 39.94% of the effect of living alone on depressive symptoms, whereas health status and social relationships accounted for only 1.27% and 5.91%, respectively.

The findings of studies conducted in the United States, New Zealand, and European countries (such as Sweden and Norway) where older adults are financially secure with public pensions and asset income suggest that living alone has become a viable lifestyle [33,34,35]. Furthermore, previous authors have suggested that living alone is unnecessarily associated with increased risk of psychological distress [13], showing that older women living alone were happy, enjoyed their independence, and were psychologically well [36]. Our finding of a large XF of household income for the negative relationship between living alone and depressive symptoms partially supports the studies conducted in the United States and Europe. Our findings suggest that without financial difficulties, living alone is not perceived as stressful and may, in fact, offset the development of depressive symptoms. Nevertheless, living alone may have a different meaning for older widows in Korea than for those living in the United States and European countries. We found that widows living alone were more likely to have a lower equivalent household income than those living with others (Table 1). It may be that older widows who are struggling under economic strain and poverty are forced to live by themselves without sufficient support from their impoverished adult children. A previous study found that 28.8% of impoverished adults aged 60 years and older who were living alone were working to support themselves [37]. It is not surprising to find that the financial factor was significant in accounting for depressive symptoms because the cost of managing depression is high in developed Asian countries [38]. Chronic medical illness plays a key role in affecting subjective health and functional status in older Asians [39], but it is important to note that the financial factor was more important than health status in influencing depressive symptoms in this study. According to the Korean Institute for Health and Social Affairs, the relative poverty rate of adults aged 60 years and older living alone has been on the rise in Korea since 2011 and reached 67.1% in 2016, which was the highest percentage among Organisation for Economic Co-operation and Development (OECD) countries. Many of the current cohort of older adults who were born in the Japanese colonial era did not prepare for aging and retirement because they had little education and invested their resources in their children throughout mid-life [40]. To make matters worse, these elderly adults are not eligible for the Korean national pension system [41]. Therefore, the majority of Korean widows from disadvantaged families may be forced to live alone for survival rather than for privacy and independence reasons. Specialized welfare policies, such as additional basic old-age pension, may be needed to target this population in order to address their financial vulnerabilities which may otherwise lead to poor mental health including high suicide rates.

Traditional Korean society valued older adults living with their children; however, the society is changing to one where greater value is placed on privacy and independence among young and old alike [7]. Furthermore, young Korean adults are struggling with harsh economic realities, including unemployment, low payment, and little job security because of the aging population and slowdown in economic growth. Given this cultural and social shift, the living arrangements of older adults may depend on the needs and living conditions of their adult children rather than their own needs. A recent Korean study found that a high economic status of aging parents was significantly associated with cohabitation with married or unmarried adult children because the adult children had economic difficulties [42]. We also found that among widows in the highest equivalent household income level, those living with their children or others were more likely to report depressive symptoms (14.7%) than those living alone (8.6%, Table 1). Moreover, older Korean women often choose to live with their children because they have been asked to look after their grandchildren [43,44]. The continuous burden of caring for grandchildren and performing housework in order to assist a working daughter or daughter-in-law may cause fatigue and stress in older women, which offsets the advantages of living with their adult children [44].

As expected, social relationship variables and social activity were significantly associated with depressive symptoms, but did not have a significant effect on the relationship between living alone and the prevalence of depressive symptoms. This finding is partially consistent with previous research [13] in that it revealed the importance of social support for the mental health of older widows. Of the social variables, relationship with adult children had the highest OR for depressive symptoms. This finding is consistent with that of other Korean studies showing that social ties to, or contact with, adult children was the most important social factor for the mental health of widows [44,45]. The relationship with adult children may have a greater impact on depressive symptoms than other types of social support in countries such as Japan, Taiwan, Spain, and Italy because the social networks of older adults are family-focused [46,47,48]. Additionally, this may stem from the tendency of older widows to maintain more active social ties to children, friends and relatives, whereas the presence and support of a spouse was more important for the mental health of widowers [16]. According to our previous studies, percentage of living alone is similar between widowers and widows, although the number of such widows is much larger because of longevity of women. Meanwhile, married older men have lower depressive symptoms, but they became more depressed after bereavement, compared to older women [44]. Good relationship with children reduced depressive symptoms among widowers to a greater extent than among widows [49]. Further research is needed to identify gender differences in the main determinants of depressive symptoms among widowed older adults. Widowed older adults suffering from depression may benefit from antidepressant treatment and grief therapy [50]. A new psychological intervention, using the life storybook creation process and incorporation of the use of narratives was found to reduce severity of depression in the elderly [51]. The life storybook intervention would be helpful for widows to achieve a closure of grief over the death of their spouses. 

Several limitations warrant caution when interpreting our findings. First, the cross-sectional design of our study did not allow us to draw inferences about causal relationships between the factors included in this study and depressive symptoms in older Korean widows. Longitudinal data would facilitate the exploration of causal relationships. Second, we could not consider the duration of widowhood, loneliness, and social isolation because they were not included in the LPOPS data. These factors may influence depressive symptoms among older widows. Third, this study did not report prevalence of past suicide attempts. Depression is a significant risk factor for suicide in the Asian elderly and engagement of social services could reduce suicidal risk by resolving social and financial problems [52]. Fourth, this study did not report the proportion of participants who suffered from caregiver stress before the spouse died. Many elderly individuals often suffer from depression due to caregiver stress by having looked after spouses with chronic medical diseases [53,54] and this depression often continues into widowhood. It would be interesting to assess the relationship between history of caregiver stress and risk of suffering from depression during widowhood. 

In spite of these limitations, our study which was conducted in Korea, where co-residency has long been a cultural tradition, suggests that economic resources are more important than health and social relationships for alleviating the negative impact of living alone on the development of depressive symptoms in older widows. Our findings highlight the need for policy interventions that provide various financial resources (i.e., additional elderly pension and priority of opportunity to work) in order to prevent and alleviate depressive symptoms in widows living alone. Moreover, our findings support living alone as a viable lifestyle among older Korean widows.

## 5. Conclusions

We examined the impact of living alone on depressive symptoms in older Korean widows and assessed the individual contributions of health, social ties, and socioeconomic factors to having depressive symptoms. Our findings suggest that living alone may not be perceived as stressful by older widows who are financially secure and that, in fact, living alone may prevent them developing depressive symptoms. Our findings underscore the importance of various forms of financial support for older widows living alone. 

## Figures and Tables

**Table 1:** Weighted percentage distribution of Korean widows aged 65 years or older living with others (N = 1101) or living alone (N = 1698) with no disability and the prevalence of depressive symptoms according to health status, social ties, and socioeconomic status.

	Co-Residency	Alone	*p*	Depressive Symptoms (%)		*p*
	N (%) or Mean (SD)	N (%) or Mean (SD)		Co-Residency	Alone	
*N*=	1101	1698		1101	1698	
*Prevalence of depressive symptoms*				20.6	24.3	
Age	75.68 (6.86)	75.73 (6.10)			**	
65–74	519 (47.1)	729 (42.9)	0.000	18.1	21.4	0.561
75–84	439 (39.9)	818 (48.2)		23.5	25.2	
85+	143 (13.0)	151 (8.9)		21.1	34.2	
Area of residence						
Urban	896 (81.4)	1230 (72.4)	0.000	20.2	24.6	0.068
Rural	205 (18.6)	468 (27.6)		22.5	23.7	
Religion						
No	243 (22.1)	514 (30.3)	0.000	23.5	27.2	0.025
Yes	858 (77.9)	1184 (69.7)		19.8	23.1	
Number of chronic diseases	(2.69, 1.69)	(2.98, 1.86)		**	**	
None	92 (8.4)	104 (6.1)	0.063	9.8	11.5	0.493
Yes	1009 (91.6)	1594 (93.9)		21.7	25.2	
Relationship with children				**	**	
Good	683 (62.0)	1042 (61.4)	0.000	12.9	14.9	0.054
Fair/poor	410 (37.2)	595 (35.0)		33.5	40.4	
No children	8 (0.7)	61 (3.6)		25.0	29.5	
Relationship with friends/neighbors				**	**	
Good	567 (51.5)	994 (58.5)	0.000	15.0	17.4	0.275
Fair/poor	534 (48.5)	704 (41.5)		26.8	34.1	
Social participation				**	**	
Yes	360(32.7)	616 (36.3)	0.056	12.5	13.6	0.918
No	741(67.3)	1082 (63.7)		24.6	30.4	
Education				**	**	
High school+	114 (10.4)	200 (11.8)	0.418	13.0	20.0	0.368
Middle school	469 (42.6)	693 (40.8)		18.2	20.5	
Primary school	518 (47.0)	806 (47.4)		24.5	28.7	
Working				**	**	
Yes	193 (17.5)	412 (24.3)	0.000	9.8	15.7	0.010
No	908 (82.5)	1286 (75.7)		22.9	27.1	
Equivalent household annual income ^a^	1932.46 (1271.95)	946.26 (924.62)		**	**	
Highest 33.3%	693 (62.9)	243 (14.3)	0.000	16.9	8.6	0.000
Middle 33.3%	301 (27.3)	631 (37.2)		25.6	20.3	
Lowest 33.3%	108 (9.8)	824 (48.5)		31.5	32.0	

^a^ Annual household income was divided by the square root of the number of household members and equivalent household income was divided into tertiles. ** *p* < 0.01 for differences among different levels of each variable. *p*-values for differences between widowed women living with others and those living alone.

**Table 2:** The explained fractions (XF) ^a^ of the health, social ties, and socioeconomic status calculated from odds ratios (95% confidence intervals [CI]) for depressive symptoms in elderly Korean widows living alone.

	Model 1	Model 2	Model 3	Model 4	Model 5	Model 6	Model 7	Model 8	Model 9
	OR (95%CI)	OR (95%CI)	OR (95%CI)	OR (95%CI)	OR (95%CI)	OR (95%CI)	OR (95%CI)	OR (95%CI)	OR (95%CI)
Living arrangement									
Coresidency									
Living alone	1.24 (1.03–1.49)	1.22 (1.01–1.47)	1.24 (1.02–1.51)	1.31 (1.09–1.58)	1.30 (1.08–1.57)	1.26 (1.04–1.51)	1.28 (1.07–1.55)	0.74 (0.60–0.93)	0.99 (0.78–1.26)
Chronic disease									
None									
Yes		2.57 (1.61–4.09)							2.56 (1.58–1.16)
Relationship with children									
Good									
Fair/poor			3.78 (3.13–4.57)						3.1 (2.52–3.80)
No children			2.45 (1.43–4.20)						1.75 (1.00–3.07)
Relationship with friends/neighbors									
Good									
Fair/poor				2.32 (1.93–2.79)					1.36 (1.10–1.66)
Social participation									
Yes									
No					2.53 (2.03–3.14)				1.95 (1.53–2.47)
Education									
High school +									
Middle school						1.17 (0.84–1.62)			0.99 (0.69–1.41)
Primary school						1.73 (1.25–2.38)			1.15 (0.80–1.64)
Working									
No							2.11 (1.63–2.72)		2.13 (1.63–2.79)
Yes									
Equivalent household income ^b^									
Highest 33.3%									
Middle 33.3%								1.87 (1.45–2.42)	1.62 (1.23–2.12)
Lowest 33.3%								3.25 (2.47–4.27)	1.93 (1.43–2.60)
Adjusted *R*^2^	0.009	0.02	0.113	0.054	0.05	0.021	0.029	0.05	0.184
XF		1.27	−0.37	−5.91	−4.92	−1.52	−3.73	39.94	19.79

Model 1 was adjusted for age, area of residence, and religion; Model 2 was adjusted for the same variables as Model 1 and chronic disease; Model 3 was adjusted for the same variables as Model 1 and relationship with children; Model 4 is adjusted for the same variables as Model 1 and relationship with friends and neighbors; Model 5 was adjusted for the same variables as Model 1 and social participation; Model 6 was adjusted for the same variables as Model 1 and education; Model 7 was adjusted for the same variables as Model 1 and working status; Model 8 was adjusted for the same variables as Model 1 and equivalent annual household income; and Model 9 was adjusted for all variables. ^a^ The XF of health status, social ties, and socioeconomic status differential odds of “depressive symptoms” for living alone and co-residency was calculated using the formula: ([OR_(model 1)_ − 1] − [OR_(model 2,3,4,5,6,7,8)_ − 1])/(OR_model1_ − 1). ^b^ Annual household income was divided by the square root of the number of household members and equivalent household income was divided into tertiles.
